# Potentially effective therapy of heavy menstrual bleeding with an oestradiol-nomegestrol acetate oral contraceptive: a pilot study

**DOI:** 10.1186/s40814-017-0130-2

**Published:** 2017-04-10

**Authors:** Edith Weisberg, Kevin McGeehan, Jane Hangan, Ian S Fraser

**Affiliations:** 1 Family Planning NSW, 328-336 Liverpool Road, Ashfield, Sydney, NSW 2131 Australia; 2 University of Sydney, Sydney, Australia; 3 University of New South Wales, Sydney, Australia

**Keywords:** Survey, Menstruation, Heavy menstrual bleeding (HMB), Menstrual pain, Quality of life (QoL)

## Abstract

**Background:**

Heavy menstrual bleeding (HMB) exceeding 80 mL per cycle leads to considerable adverse impact on a woman’s iron metabolism, incidence of iron deficiency and anaemia, as well as her functioning in society.

The objective of the study is to determine the potential efficacy of a Monophasic oestradiol-17β-nomegestrol acetate (E_2_/Nomac) combined oral contraceptive pill on measured menstrual blood loss as a pilot study in 12 women with objectively demonstrated HMB (>80 mL per cycle). The pilot study aimed to recruit 20 women.

**Method:**

Consented women completed the HMB questionnaire. The blood was taken for haemoglobin, transferrin, iron saturation, TIBC, serum iron and ferritin. Women were given verbal and written detailed instructions for MBL collection for three control cycles and four treatment cycles.

**Results:**

Forty-three women were enrolled, but 31 were ineligible and withdrawn (mainly for failure to meet eligibility criteria). Twelve women entered the treatment phase and commenced the E_2_/nomegestrol acetate (NOMAC) 24/4 combined pill treatment on the first day of their fourth cycle. All women with complete MBL measurements had >50% reduction in MBL on treatment (exact 95% confidence interval for proportion with MBL reduction >50%: 69 to 100%). The mean percent reduction in MBL between pretreatment and during treatment was 76.9%, and the median was 79% with a range of 53.7 to 100%.

**Conclusions:**

This pilot study indicates that the E_2_/NOMAC COC will provide a useful potential option for treating HMB in women with FIGO classification AUB-E (primary endometrial causes) but requires a larger placebo-controlled study for confirmation.

## Background

The widely accepted research definition of heavy menstrual bleeding (HMB) is menstrual blood loss exceeding 80 mL per menstrual period [1]. Regular blood loss of this magnitude leads to considerable adverse impact on a woman’s iron metabolism, incidence of iron deficiency and anaemia, as well as general health issues and her functioning in society [2]. The impact HMB has on a woman’s quality of life (QoL) can be a significant motivation for seeking medical help. Most sufferers seek simple measures for management and may initially avoid attendance at general practitioners or medical specialists [1–3]. Many have concern that major surgery may be required to manage their problem, and wish to avoid this.

HMB accounts for considerable morbidity in women of reproductive age and can greatly affect many aspects of their health and quality of life. This includes problems of length and heaviness of bleeding, severity of menstrual pain, problems with flooding and clots, problems with soiling, being confined to bed, mood changes, severe lethargy and fatigue, anxiety, psychiatric morbidity, iron deficiency and anaemia [4–11]. Around half of all women presenting with HMB have no evidence of underlying structural pelvic pathology (i.e. these are women with AUB-E (primary endometrial), AUB-O (primarily ovulatory disturbances) or AUB-C (coagulopathies) according to the FIGO PALM-COEIN classification of causes of HMB [12]). These women may be particularly suitable for medical treatment with only limited initial investigations.

Epidemiological evidence has suggested that as many as 52% of all women at some point exhibit HMB symptoms. A recent survey conducted in five European countries found that among 1225 women, 27% of the respondents currently exhibited at least two predetermined symptoms suggestive of HMB [13]. Many women affected by HMB do not seek medical help, and few of those who do consult physicians report that they have received appropriate effective treatment. HMB continues to be under-diagnosed and poorly treated [13]. There is a real need for highly effective and simple measures. A novel combined oral contraceptive pill (COCP) with simple regimen and highly effective HMB suppression is greatly needed.

Objective or semi-objective measurement of blood loss is not practical in routine clinical practice; therefore, clinical evaluation of the symptom of HMB relies heavily on self-reporting, reflecting the subjective experience of individual women in the absence of any absolute reference point. Therefore, there has been a move recently to define HMB in clinical practice settings in terms of its impact on a woman’s physical, emotional, social and material quality of life rather than on any current objective measure [6]. This means that, in addition to management of iron deficiency, any active therapy must also aim for improvement of QoL measures.

In women with no underlying structural pathology, medical therapy is considered the preferred treatment for HMB [14]. However, in order to determine the efficacy of a new treatment, it is necessary to measure the actual physical monthly blood loss both prior to and during treatment. In order to achieve reliable measurement of menstrual blood loss, women are required to collect all their soiled sanitary protection as well as ensure none is lost into the toilet or shower [15, 16]. This is extremely onerous especially as most women are now in full time work and must continue their menstrual blood collections at their workplace, a prospect that many women find daunting. This contributes to making modern-day recruitment of subjects difficult.

The main objective of this open-label pilot study was to determine the potential efficacy of a monophasic oestradiol-17β-nomegestrol acetate (E_2_/Nomac) combined oral contraceptive pill on measured menstrual blood loss in 12 women with objectively demonstrated HMB (greater than 80 mL of blood loss per cycle).

A secondary objective was to determine how feasible it would be to recruit for a larger placebo-controlled study of this oestradiol-17β-based COCP treatment, if the treatment appeared to be efficacious.

## Methods

### Inclusion criteria

Women aged 18–50 who demonstrated heavy menstrual blood loss (MBL) greater than 80 mL in at least two of three regular menstrual periods during the pre-treatment phase and no significant uterine pathology on pelvic ultrasound were eligible to enter the treatment phase of the study. Hence, these women were likely to fit into categories AUB-E, and perhaps AUB-C, of the FIGO PALM-COEIN classification of causes [12]. Eligible women were only excluded if they had contraindications to combined oral contraceptives. Ethics approval was obtained from the Family Planning NSW Ethics Committee (Ethics Committee application reference number R2012-07).

### Recruitment

Women were recruited from the Sydney Centre for Reproductive Health Research database, Facebook and radio advertisements. Women who identified themselves as having HMB were telephone screened by an experienced research assistant. If their responses indicated that they were likely to have objective HMB, the Research Coordinator (RC) discussed the MBL study. Interested women were sent written information about the study and contacted 1 week later. If willing to participate, an appointment was made to attend the Research Centre where the RC provided study details and obtained written informed consent.

### Study procedures

#### Run-in period

Women who had given consent completed the online HMB questionnaire [17] (attachment 1), a detailed validated questionnaire looking at various aspects of quality of life before entering and on completion of the study [17]. Blood was taken in standard manner for haemoglobin estimations, transferrin saturation, serum iron and ferritin. Women were given verbal and written detailed instructions for MBL collection and provided with cotton pads, tampons and wipes suitable for MBL estimations, collection bags, cold packs and cold boxes. Participating women were asked to collect all used sanitary protection for three menstrual periods without treatment (cycles 1, 2 and 3) and complete a paper diary entering the details of their bleeding and any symptoms that they experienced, as well as the use of all sanitary protection. Treatment with oestradiol-17β 1.5 mg–nomegestrol acetate 2.5 mg monophasic COC tablets on a 24 + 4 day regimen (MSD/Merck; marketed in Australia as Zoely) was started on day 1 of the next period (cycle 4). This menstrual period (cycle 4) was not included in the formal analysis of the following three ‘during treatment’ cycles (cycles 5, 6 and 7).

In addition to the paper diary, recording days of bleeding and number of tampons and pads used each day, they also completed a chart on which they indicated the size of any ‘clot’ passed, the amount of blood that was lost in the toilet according to the icon on the chart, any blood lost in staining of undergarments, etc.

All tampons and pads used for each of three consecutive menses were sent at the end of each menses by courier to the Queen Elizabeth II Research Institute for Mothers and Infants for the MBL estimations, which were carried out by the alkaline haematin method, which has been applied in research studies in our laboratory for over 35 years [18]. All costs of sanitary protection, transport materials and couriers were covered by the project.

### Sample size and analysis

The primary efficacy endpoint was the proportion of women with a reduction ≥50% from baseline in objectively measured menstrual blood loss. Secondary efficacy endpoints were the number and proportion of cycles with a normal blood loss (≤80 mL, and the total group reduction in measured blood loss).

We planned to recruit 20 women to the pilot study. Based on results of previous studies with ethinyl-oestradiol-based combined oral contraceptives, we assumed that 50% of the women would achieve a reduction ≥50% in mean blood loss. With 20 women in the study, the resulting confidence interval for this percentage has a precision of ±22%. This pilot study would then indicate that the percentage of women with a complete response would be at least 28%. A sample size of 20 would also have a power greater than 80% to detect a reduction of 100 mL in objectively measured MBL over a 3-month reference period assuming *α* = 0.05 and standard deviation of 100 mL (based on studies with the oestradiol-valerate–dienogest COCP, Qlaira, Bayer Healthcare). ‘Qlaira’ is the only other oestradiol-17β-based COCP studied for its effect on HMB.

The mean of continuous measures of MBL and iron metabolism parameters were calculated for the three cycle pre-treatment phase (cycles 1, 2 and 3) and the ‘during treatment phase’ (cycles 5, 6 and 7). We then calculated the percent reduction in MBL by comparing the mean for the pre-retreatment phase with that for the treatment phase.

The continuous measures of MBL and iron metabolism parameters were analysed using the signed-rank test due to the small sample size and expected skewness of the measures. Categorical responses to questions before and during treatment were compared using McNemar’s test for paired proportions.

We calculated an exact 95% confidence interval for the primary outcome and proportion of women with a reduction ≥50% from baseline in MBL. The difference between pre-treatment and during treatment in continuous measures of MBL and the iron metabolism parameters were analysed using the signed-rank test due to the small sample size and expected skewness of the measures. *t* tests and 95% confidence intervals of the difference in the means of these continuous measures were also calculated. Categorical responses to questions before and after treatment were compared using McNemar’s test for paired proportions. All analyses were carried out using SAS v9.3 (SAS Institute).

## Results

**Fig. 1:**
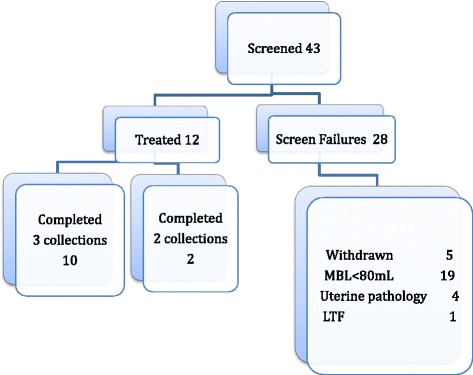
Study flow chart

Forty-three women, mean age 35.7 years (range 20–48), were enrolled in this pilot study, of whom 31 either withdrew or were ineligible due to underlying pathology or otherwise not complying with entry criteria (Fig. 1). Twelve women entered the treatment phase and commenced the E_2_/nomegestrol acetate 24/4 combined pill treatment on the first day of their next menses and continued treatment for three cycles. Ten women continued to collect their used sanitary protection for the following four cycles (including the three full-treatment cycles 5, 6 and 7) and send them by courier to the University of Sydney as previously described. One subject was discontinued after taking two cycles of E-17β-Nomac because of a significant rise in blood pressure and therefore did not collect the seventh menstrual period. Another subject did not collect in menstrual period 5 as she was travelling overseas. Their results are reported separately.

All women showed considerable variation in MBL during their three run-in periods (Table 1). On average, 70% of individual cycles had MBL greater than 80 mL. This meant that only 30% of pretreatment menstrual periods had an MBL of <80 mL while 97% of periods during the treatment phase were less than 80 mL. All women with complete MBL measurements had greater than 50% reduction in MBL on treatment (exact 95% confidence interval for proportion with MBL reduction greater than 50%: 69 to 100%).

**Table 1:** Individual subject’s measured menstrual blood loss (mL) per study cycle

	Pretreatment			COCP start		Treatment cycles	
Subject	Cycle 1 (mL)	Cycle 2 (mL)	Cycle 3 (mL)	Cycle 4 (mL)	Cycle 5 (mL)	Cycle 6 (mL)	Cycle 7 (mL)
1	99.5	60.8	128.7	56.6	9.9	24.5	Discontinued
2	73.9	208.4	150.0	35.1	18.9	16.9	25.3
3	42.1	318.3	79.4	28.7	14.7	17.2	28.7
4	87.6	65.0	65.0	92.8	33.8	41.9	25.0
5	127.6	83.6	131.0	194.3	0	27.7	18.1
6	280.9	88.4	58.6	80.4	33.2	42.0	61.5
7	259.1	222.7	244.4	109.0	60.4	13.3	96.8
8	92.8	60	109.5	40.9	6.4	21.6	20.5
9	84.0	130.4	32.9	84.6	6.4	38.3	31.4
10	230.5	104.4	169.6	189.1	0	0	0
11	92.5	212.3	218.8	170.2	Not collected overseas	51.2	61.2
12	137.4	23.0	92.3	47.0	43.5	24.2	29.4

Started E_2_/NOMAC on the first day of bleeding in this cycle

**Fig. 2:**
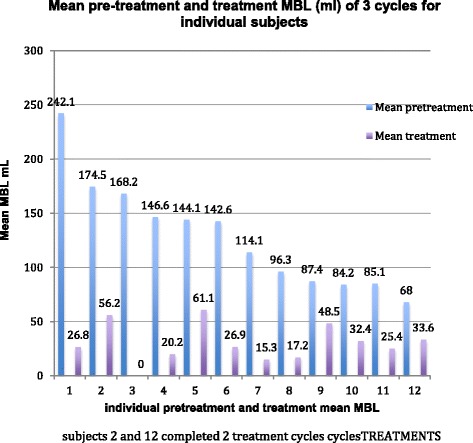
Mean paired pretreatment MBL for three pre-treatment cycles and three cycles on treatment with E_2_/NOMAC for 12 individual women

The mean percent reduction in MBL between pretreatment and during treatment cycles was 76.9% and the median was 79% (with a range of 53.7 to 100%) (Table 2). The two subjects with only two post-treatment collections also had 81.2 and 79.9% reduction in MBL respectively. The median absolute reduction in monthly blood loss was 98 mL per period per subject with a range of 39–185 mL. Individual reductions in MBL between pre- and during treatment are shown in Fig. 2. In 33 of 34 treatment cycles, blood loss had reduced to within the normal range (<80 mL).

**Table 2:** Comparison of mean and median monthly reduction in MBL for the group between the pre-treatment phase and the treatment phase

Variable	Number	Mean (mL)	Std Devn (mL)	Median (mL)	Minimum (mL)	Maximum (mL)
Mean (mL) before treatment (cycles 1, 2 and 3)	10	128.4	52.0	128.4	72.5	242.1
Mean (mL) on treatment (cycles 5, 6 and 7)	10	26.6	16.3	22.9	0.0	56.8
Difference in means (before treatment compared to on-COC)	10	101.9	49.3	97.9	39.0	185.2
Percent reduction	10	76.9	13.8	79.0	53.7	100.0

Ninety-five percent confidence interval for difference in means (66.6, 137.1), *P* = 0.0001 (paired *t* test), *P* = 0.002 (signed-rank test). Ninety-five percent confidence interval for percent reduction (67.0, 86.8), *P* < 0.0001 (one sample *t* test), *P* = 0.002 (signed-rank test)

### Effect of treatment on quality of life

There was no statistically significant difference in reported QoL during treatment in regard to pain, effect on leisure activities or effect on sex life in those women who had sexual relationships during menstruation.

The only significant change shown in iron parameters was an increase in mean ferritin levels (Table 3).

**Table 3:** Iron study results before commencing treatment and on completion of treatment

	Number	Mean	95% confidence interval	Median	Minimum	Maximum	*P* value (*t* test)	*P* value (signed rank)
HB before treatment	10	135.1		134.0	123	148		
HB end of treatment	10	137.0		136.5	125	150		
Difference (on-before)	10	1.9	−3.1, 6.7	3.5	−11	12	0.47	0.56
Transferrin before treatment	10	4.91		3.05	2.4	23		
Transferrin on treatment	10	4.82		2.85	2.3	23.3		
Difference (on-before)	10	−0.09	−0.25, 0.07	−0.05	−0.6	0.3	0.24	0.28
TIBC before treatment	10	64.2		66	54	76		
TIBC on treatment	10	62.4		63	52	72		
Difference (on-before)	10	1.8	−1.5, 5.1	1	−6	12	0.24	0.34
*T*-saturation before treatment	10	0.297		0.32	0.11	0.49		
*T*-saturation on treatment	10	0.268		0.285	0.09	0.4		
Difference (on-before)	10	−0.029	−0.128, 0.070	−0.095	−0.15	0.2	0.53	0.54
Ferritin before treatment	10	28.1		27	7	44		
Ferritin on treatment	10	41		45.5	17	63		
Difference (on-before)	10	12.9	5.9, 19.9	12.5	0	32	*0.002*	*0.004*

A value of *P* <0.05 was taken as significant. This includes all values in italics

## Discussion

The E_2_/Nomac combined pill was extremely efficient at significantly reducing MBL in all 12 women with objectively measured HMB. The treatment drug produced a 50% or greater reduction in MBL in virtually all women. In all but one treated cycle blood loss was well below the established upper norm of 80 mL. If extrapolated over a 3-month (three menstrual periods) reference period, this would have resulted in an overall total menstrual blood loss volume reduction of 305.4 mL “saved” for each woman every 3 months during E_2_/Nomac treatment or a gross total around 1222 mL “saved” annually. This demonstrates a huge additional requirement for iron intake per year by these women when they are without treatment.

It was surprising that the post-treatment questionnaire found that our E_2_/Nomac treatment, which reduced blood loss to well below the upper limit of normal, had little effect on the subject’s QoL compared to pretreatment, possibly due to the small number of subjects and the limited duration of the study. The results suggest that pain with menstruation was not significantly ameliorated, although pain scores were not specifically investigated. A trend for improvement in coping with leisure activities and sex life was also apparent, although not statistically significant.

There was a significant increase in serum ferritin levels following treatment but no other significant change in iron parameters. The improvement in ferritin levels clearly reflects the very significant reduction in MBL for all subjects. The failure of the other iron indices to show a significant difference probably relates to the short 3-month treatment period and the small numbers in this pilot study. However, caution needs to be used in interpretation of any hypothesis testing with such small numbers.

The pre-treatment questionnaire indicated that 50% of women with demonstrated HMB experienced a significant adverse effect on QoL parameters during menstruation. An efficient medical treatment which significantly reduces blood loss (to a greater degree than any other oral therapy) and has minimal side effects is an important addition to the range of therapies available for managing the HMB problem.

Our result of a 79.6% mean reduction in MBL compared very favourably with other studies which also used objective quantification of MBL during medical treatments of HMB. A number of studies have reported on the effect of combined oral contraceptives (COCP) on HMB. Only four have investigated the efficacy of COCPs using objectively measured MBL [18–21]. The first randomised study demonstrated a mean reduction of 43% in MBL over two cycles of treatment with a 30-μg EE COCP in 12 women [18]. The second larger randomized study of 56 women reported a 35% mean reduction in MBL over 12 months of treatment with a 30-μg EE COCP [19]. An early, non-randomised trial in 1971 of 164 women [20], which also studied objective measurement of MBL, found that a high-dose (50–75 μg EE) COCP reduced mean MBL by 52.6%. The fourth study which trialled a dienogest/estradiol valerate combined pill (this was the first HMB study with an oestradiol-17β-based COCP) in a much larger study (*n* = 231) over 12 months reported a mean reduction of MBL of 69.4% (median 79.2%) [21] only slightly less than in the present trial with E_2_/Nomac. This present E_2_/Nomac study showed a mean reduction of 76.9% (median 79.0%). These two studies suggest that oestradiol-17ß-based COCs may be in a separate COCP class with respect to their therapeutic benefit on HMB. It is probable that oestradiol-17β combined with either Nomac or dienogest has a more favourable response on the endometrium in producing menstrual period haemostasis than ethinyl-oestradiol combinations.

A previous contraceptive study of women without HMB treated with E_2_/Nomac found that 30% had completely absent withdrawal bleeding at the end of 12 months [22]. In contrast, a study comparing a COCP using a 24/4 regimen with the same COCP on a 21/7-day regimen [23] found no difference in bleeding patterns suggesting that the improvement in HMB in our study was not due to the 24/4 regimen but resulted from the oestradiol-17β-based formulation of the COCP.

This present study was a pilot trial to ascertain whether combining oestradiol-17β with nomegestrol acetate in a monophasic regimen would be effective in reducing MBL in women with AUB-E with objectively confirmed HMB. The main weakness lies in the small number of women we were able to recruit with objective HMB caused by AUB-E, due to changing social and cultural dynamics. E_2_/Nomac may be as effective in reducing MBL as the oestradiol valerate/dienogest combination, but further studies in larger numbers of women will be necessary to confirm this. Hypothesis testing is not possible with limited numbers of subjects. Larger studies may well also run into problems of recruitment of large numbers of well-investigated women, due to general reluctance of both investigators and women to collect all used sanitary protection. Although pictorial blood loss assessment charts are less accurate in objective measurement of menstrual blood loss than in alkaline haematin method, they are accurate enough for sound comparison of pre-and intra-treatment assessments, subject to meticulous care with collections and assessment by the same investigator. This would be a less expensive option, and recruitment is likely to be easier. We would suggest that any future trial should ideally compare, in a randomized manner, this E2/Nomac combination with a widely used 30-μg ethinyl-oestradiol-containing combination. A total of 50 enrolled women with objectively measured menstrual blood loss studied over three pre-treatment months and a minimum of three intra-treatment months will offer substantially increased power compared with the present study. This should be sufficient to test the hypothesis that the E2-based COCs are more effective treatments for HMB than for ethinyl-oestradiol-based COCs.

## Conclusions

This pilot study indicates that the E_2_/Nomac COCP is likely to provide a useful new option for treating HMB in women who have probable primary endometrial causes of the abnormal bleeding (AUB-E). This effect may be greater than that seen with women using ethinyl-oestradiol-based COCPs.

## References

[1] Fraser IS, Langham S, Uhl-Hochgraeber K (2009). Health-related quality of life and economic burden of abnormal uterine bleeding. Expert Rev Obstet Gynecol.

[2] Lethaby A, Farquhar C (2003). Treatments for heavy menstrual bleeding. BMJ.

[3] Fraser IS, Critchley HO, Munro MG, Broder M (2007). A process designed to lead to international agreement on terminologies and definitions used to describe abnormalities of menstrual bleeding. Fertil Steril.

[4] Byles JE, Hanrahan PF, Schofield MJ (1997). ‘It would be good to know you’re not alone’: the health care needs of women with menstrual symptoms. Fam Pract.

[5] Santer M, Wyke S, Warner P (2007). What aspects of periods are most bothersome for women reporting heavy menstrual bleeding? Community survey and qualitative study. BMC Women's Health.

[6] Shapley M, Jordan K, Croft PR (2003). Increased vaginal bleeding: the reasons women give for consulting primary care. J Obstet Gynaecol.

[7] Warner PE, Critchley HO, Lumsden MA (2004). Menorrhagia II: is the 80-mL blood loss criterion useful in management of complaint of menorrhagia?. Am J Obstet Gynecol.

[8] Carlson KJ, Miller BA, Fowler FJ (1994). The Maine Women’s Health Study: II. Outcomes of nonsurgical management of leiomyomas, abnormal bleeding, and chronic pelvic pain. Obstet Gynecol.

[9] Coulter A, Peto V, Jenkinson C (1994). Quality of life and patient satisfaction following treatment for menorrhagia. Fam Pract.

[10] Ruta DA, Garratt AM, Chadha YC (1995). Assessment of patients with menorrhagia: how valid is a structured clinical history as a measure of health status?. Qual Life Res.

[11] Marshall J (1998). An exploration of women’s concerns about heavy menstrual blood loss and their expectations regarding treatment. J Reprod Infant Psychol.

[12] Munro MG, Critchley HO, Broder MS (2011). FIGO classification system (PALM-COEIN) for causes of abnormal uterine bleeding in nongravid women of reproductive age. Int J Gynaecol Obstet.

[13] Fraser IS, Mansour D, Breymann C (2015). Prevalence of heavy menstrual bleeding and experiences of affected women in a European patient survey. Int J Gynecol Obstet.

[14] Haththotuwa R, Goonewardene M, Desai S (2011). Management of abnormal uterine bleeding in low-and high-resource settings: consideration of cultural issues. Semin Reprod Med.

[15] National Collaborating Centre for Women's and Children's Health (2007). National Institute for Health and Clinical Excellence (NICE) clinical guidance 44: heavy menstrual bleeding.

[16] Bulmer P (2008). Menorrhagia. Obstet Gynaecol Reprod Med.

[17] Weisberg E, McGeechan K Fraser IS. Effect of perceptions of menstrual blood loss and menstrual pain on women’s quality of life. Eur J Contracept Reprod Health Care. 2016;431-435. [Epub ahead of print] 10.1080/13625187.2016.1225034 27623183

[18] Fraser IS, Weisberg E, Minehan E, Johansson EDB (2000). A detailed analysis of menstrual blood loss in women using Norplant and Nestorone progestogen-only contraceptive implants or vaginal rings. Contraception.

[19] Fraser IS, McCarron G (1991). Randomized trial of 2 hormonal and 2 prostaglandin-inhibiting agents in women with a complaint of menorrhagia. Aust N Z J Obstet Gynaecol.

[20] Shabaan MM, Zakherah MS, El-Nashar SA, Sayed GH (2011). Levonorgestrel-releasing intrauterine system compared to low dose combined oral contraceptive pills for idiopathic menorrhagia: a randomized clinical trial. Contraception.

[21] Nilsson L, Rybo G (1971). Treatment of menorrhagia. Am J Obstet Gynecol.

[22] Fraser IS, Römer T, Parke S (2011). Effective treatment of heavy and/or prolonged menstrual bleeding with an oral contraceptive containing estradiol valerate and dienogest: a randomized, double-blind phase III trial. Hum Reprod.

[23] Christin-Maitre S, Laroche E, Bricaire L (2013). A new contraceptive pill containing 17β-estradiol and nomegestrol acetate. Women's Health.

